# A case of atypical thymic carcinoid mimicking a paraganglioma

**DOI:** 10.1016/j.ijscr.2019.11.016

**Published:** 2020-01-14

**Authors:** Motoaki Yasukawa, Tomoko Uchiyama, Takeshi Kawaguchi, Noriyoshi Sawabata, Chiho Ohbayashi, Shigeki Taniguchi

**Affiliations:** a Department of Thoracic and Cardiovascular Surgery, Nara Medical University School of Medicine, Kashihara, Nara, Japan; b Department of Diagnostic Pathology, Nara Medical University School of Medicine, Kashihara, Nara, Japan

**Keywords:** MS, median sternotomy, TET, thymic epithelial tumour, PET/CT, positron emission tomography/computed tomography, MIT, minimally invasive thoracotomy, OT, open thoracotomy, NET, neuroendocrine tumour, CT, computed tomography, SUVmax, maximum standardised uptake value, HPFs, high-power fields, Neuroendocrine tumour, Atypical carcinoid, Thymic epithelial tumour

## Abstract

**Background:**

Thymic atypical carcinoid has high recurrence and metastasis rates due to frequent lymph node metastases. The aim of the study is to report a case of atypical thymic carcinoid mimicking a paraganglioma and to further explain the benefits of using median sternotomy (MS) approach even in thymic epithelial tumours (TETs) sized less than 5 cm.

**Case presentation:**

The patient was a 59-year-old asymptomatic man. During a medical check-up, positron emission tomography/computed tomography (PET/CT) showed a 4.5 cm-diameter thymus with remarkable uptake. Thoracic surgery was performed to completely remove the tumour with lymph node dissection using MS because of possible malignancy. Although MS is accepted as the standard approach for TETs, minimally invasive thoracotomy (MIT) has emerged over recent decades. Maintaining surgical safety is priority; MIT is generally selected in <5-cm-diameter tumours. Here, we considered that the tumour could be resected using MIT. However, because PET/CT showed marked uptake, we selected the MS approach. Thus, MS can be applied even for small-sized TETs.

**Conclusion:**

Thymic atypical carcinoid should be considered when PET/CT shows high-uptake tumours in the anterior mediastinum. Clinicians should consider using the MS approach, even if the tumour is <5 cm.

## Highlights

• Preoperative diagnosis of thymic epithelial tumours is challenging. Atypical carcinoid and paraganglioma are similar-appearing.
• Thymic atypical carcinoid has high recurrence and metastasis rates due to frequent lymph node metastases.
• Thymic atypical carcinoid should be considered when positron emission tomography/computed tomography shows high-uptake tumours.
• Clinicians should consider using the Median sternotomy approach, even if the tumour is <5 cm.

## Graphical abstract

**Figure:**
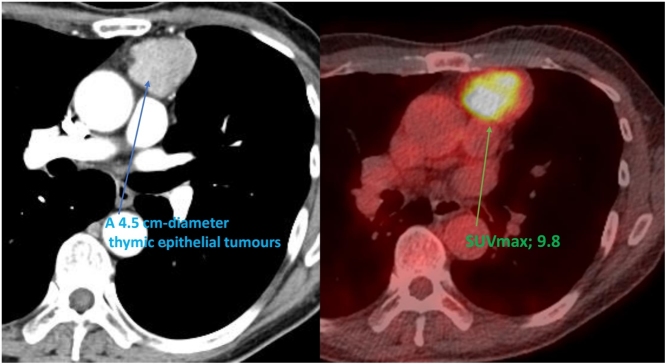

## 1 Introduction

For thymic epithelial tumour (TET) resection, minimally invasive thoracotomy (MIT) has been proposed as an alternative to conventional open thoracotomy (OT) via median sternotomy (MS) [1]. Primary neuroendocrine tumours (NETs) of the mediastinum are rare. Atypical thymic carcinoid belongs to the intermediate grade of NETs. Overall 5- and 10-year survival rates are 56–77% and 30%, respectively [2]. Complete resection is the most effective treatment and an important prognostic factor [3]. Because of the aggressive nature of thymic carcinoids and frequency of lymph node metastasis, several reports have recommended complete resection with lymph node dissection, although systemic nodal resection is yet to be standardised [4]. In general, to resect TETs, MIT or OT is selected based on tumour size reference that guarantees an oncological safe tumour resection. In the present case, considering the tumour size, its less invasive nature using computed tomography (CT), and a diagnosis of non-myasthenic thymoma, complete resection could have been performed using MIT. However, positron emission tomography/CT (PET/CT) showed marked uptake; therefore, we performed MS for en bloc resection with lymph node dissection. Here, we report a case of primary atypical thymic carcinoid mimicking paraganglioma.

This work has been reported in line with SCARE criteria [5].

## 2 Case presentation

**Fig. 1:**
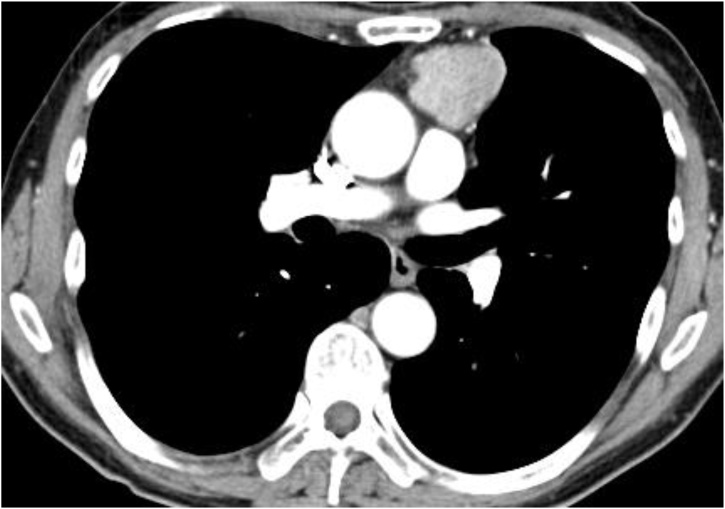
(A, B) Chest computed tomography. A 4.5-cm-diameter thymic tumour.

**Fig. 2:**
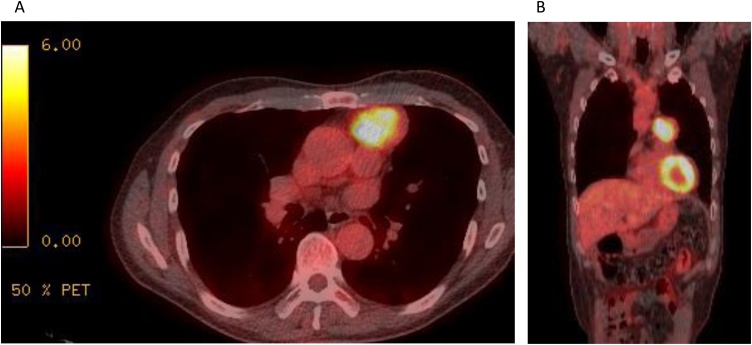
(A–C) Positron emission tomography/computed tomography. The thymic mass showing marked uptake of 18F-fluorodeoxyglucose; the early maximum standardised uptake value (SUVmax) of the mass was 9.8.

A 59-year-old man was referred to our hospital for the appearance of an abnormal shadow on a chest roentgenogram during a medical check-up. He had no symptoms and no history of neuroendocrine disorder. Physical and laboratory test findings were normal. Chest CT showed a 4.5-cm-diameter thymic tumour (Fig. 1). PET/CT showed that the mass had marked uptake of 18F-fluorodeoxyglucose, with early maximum standardised uptake value (SUVmax) of 9.8 (Fig. 2A, B). The patient underwent total thymectomy via MS. The adhering left pleura with tumour invasion was excised. The tumour was limited to the thymus without extension to the pericardia and left hilum. Radical en bloc resection and lymph node staging were performed. Operation time was 119 min, and blood loss volume was 40 mL.

**Fig. 3:**
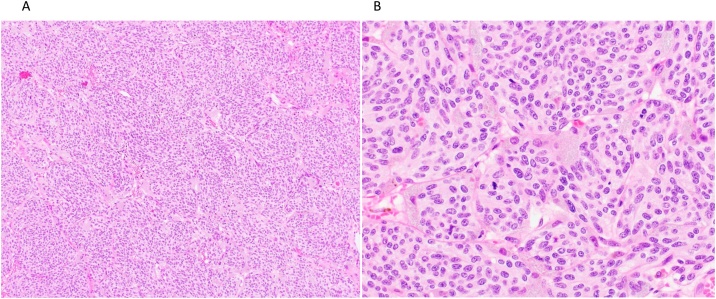
Histological findings. (A, B) Microscopic view showing that the tumour comprises spindle-shaped cells with eosinophilic cytoplasm. (Hematoxylin and eosin: A; ×10, B; ×40).

**Fig. 4:**
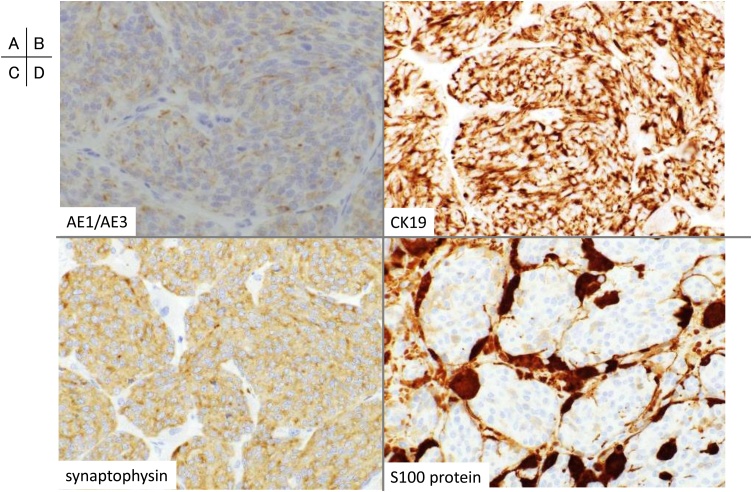
Immunohistochemical findings. The tumour cells stained positively for (A) AE1/AE3, (B) CK19, and (C) synaptophysin. The sustentacular cells stained positively for (D) S100 protein. The immunoprofile supports a diagnosis of atypical carcinoid. [(A–D: immunohistochemical staining, ×40].

Histologic examination revealed that the tumour comprised spindle-shaped cells with a pale eosinophilic cytoplasm. Necrosis was absent, and mitotic figures were 7 per 10 high-power fields (10 HPFs). The MIB-1 index was 8.5%. Fat tissue and vascular invasion was frequently observed. Initially, histopathological diagnosis was type A thymoma with atypical features (Fig. 3A, B). Immunohistochemical staining revealed cytokeratin (AE1/AE3), CK19, CD56, synaptophysin, chromogranin, and c-kit positivity and CK7 and CK20 negativity of the tumour cells. Tumour cells arranged in the nests so-called zellballen surrounded by thin fibrovascular stroma and sustentacular cells highlighted by immunostaining for S100 protein (Fig. 4). As the marker of neuroendocrine tumours and S100 protein were positive, differential diagnoses were carcinoid and paraganglioma. However, paraganglioma was ruled out because this tumour revealed positivity for cytokeratin and CK19. Although devoid of necrotic areas, mitotic rate was within 2–10 per 10 HPFs, the definitive diagnosis was atypical carcinoid of the thymus according to WHO classification of thymic neuroendocrine tumours [2]. There was no lymph node metastasis. The postsurgical course was uneventful, and he was discharged on postoperative day 8. Postoperative radiotherapy was not performed.

## 3 Discussion

Preoperative diagnosis of TETs is challenging. Although CT and magnetic resonance imaging can provide detailed information on masses, including their size, relationship to the surrounding structures, and tissue characteristics, the diagnosis relies mainly on pathological examination. Obtaining a pathological diagnosis of TETs, in addition to the clinical diagnosis, is not always easy, because this is not always technically possible and is invasive.

The National Comprehensive Cancer Network guideline [6] has suggested complete TET excision to be performed without preoperative biopsy when resectable. Complete resection is the most important factor influencing a favourable outcome [3]. Although tumour excision with total thymectomy is the mainstay of treatment, optimal mode of resection of each TET remains controversial [7].

Regarding thymic carcinoids, nodal sampling should be performed during their resection because of the aggressive nature of these tumours that have high recurrence and metastasis rates due to frequent lymph node metastases [3,4]. In addition, nodal staging may help to guide adjuvant treatment; however, systemic nodal dissection or sampling is yet to be standardised [4].

Thymectomy is the most commonly indicated for TETs. For complete resection of TETs, MS has long been the accepted standard approach [1]. During the past two decades, MIT has been proposed as an alternative to conventional OT via MS [1]. MIT guarantees oncological outcomes and is less invasive for patients with TETs. However, concerns regarding the chances of tumour capsule rupture and risk of pleural dissemination using the MIT approach have been raised. Most investigators accept that MIT is technically safe and feasible for TETs with diameters <5 cm [7]. During the last decade, MIT, including robotic thymectomy, tends to be increasing in proposal for larger tumour and more advanced stage patients [1,8]. Several reports suggested that size of thymoma was not an important consideration in the decision to proceed with MIT [1,8]. Burt et al. [1] reported that surgical approach, whether MIT or OT, was not associated with completeness of resection. Actually, if initial attempts at MIT lead to a surgical resection of TET, the surgeon might deem to resect TET completely even though he could not have completely resected pathologically. Interestingly, Friedant et al. [9] reported that the rate of conversion to OT was only 2.4%. However, patients in whom invasion of mediastinal structure is highly suspected are better served by OT. Recently, PET/CT findings provided useful information for the differential diagnosis of TETs by using the correlation between malignancy and SUVmax [10].

In this case, CT showed that tumour was not invasive and tumour size was 4.5 cm (<5 cm), whereas PET/CT showed that the tumour had a marked uptake. The preoperative diagnosis was thymoma with a high malignant potential; therefore, we performed thymectomy via MS. En bloc complete resection was performed without tumour capsule injury during the intervention. Fortunately, there was no lymph node metastasis. If we had selected the MIT approach, we might have injured the tumour capsule and disseminated the tumour; moreover, lymph node dissection could have been unsatisfactory.

For the definitive diagnosis of TETs, histological examination remains the mainstay. Paraganglioma and atypical carcinoid tumour are similar-appearing NETs. Paraganglioma is typically benign. S100 protein is important for visualising paraganglioma sustentacular cells [11]. However, Gesney et al. [12] reported that S100-positive sustentacular cells were often found in a proportion of carcinoid tumours. In this case, thymic atypical carcinoid sustentacular cells were S100 positive. Therefore, an atypical carcinoid might be misdiagnosed as a paraganglioma. Finally, the pathological diagnosis was atypical thymic carcinoid because of cytokeratin positivity. Although we performed complete tumour resection and confirmed the absence of lymph node metastasis, we followed up the patient carefully because of possible malignancy.

## 4 Conclusions

Thymic atypical carcinoid should be considered when PET/CT shows high-uptake tumours in the anterior mediastinum. Clinicians should consider using the MS approach, even if the tumour is <5 cm.

## Conflicts of interest

None.

## Sources of funding

None.

## Ethical approval

This case report is not research study, therefore approval was not given. The ethical approval has been exempted by our institution.

## Consent

We obtained written consent from the patient for publication of case report and accompanying images.

## Author contribution

MY, TK, and NS performed the operation.

MY prepared and wrote the manuscript.

TU and CO made the pathological diagnosis.

ST reviewed the manuscript.

All authors have read and approved the final manuscript.

## Registration of research studies

None.

## Guarantor

Motoaki Yasukawa.

## Provenance and peer review

Not commissioned, externally peer-reviewed.
